# Vitamin K Biochemistry and Pharmacokinetics: The Basis of Late Vitamin K Deficiency Intracranial Bleeding in Early Infancy

**DOI:** 10.3390/ijms27094000

**Published:** 2026-04-29

**Authors:** Serafina Perrone, Virginia Beretta, Vincenzo Raitano, Liana Cerioni, Silvia Carloni

**Affiliations:** 1 Neonatology Unit, Department of Medicine and Surgery, University of Parma, Pietro Barilla Children’s Hospital, 43121 Parma, Italy; serafina.perrone@unipr.it (S.P.); virginia.beretta@unipr.it (V.B.); vincenzo.raitano@ao.pr.it (V.R.); 2 Department of Biomolecular Sciences, University of Urbino Carlo Bo, Via Aurelio Saffi 2, 61029 Urbino, Italy; liana.cerioni@uniurb.it

**Keywords:** vitamin K, neonates, PIVKA-II, intracranial hemorrhage, pharmacokinetics, vitamin K deficiency, breastfeeding, coagulation, biomarkers

## Abstract

Vitamin K is a fat-soluble vitamin essential for the activation of vitamin K-dependent proteins involved in coagulation and other physiological processes. Neonates are particularly vulnerable to vitamin K deficiency due to limited placental transfer, low hepatic stores, immature liver function, and insufficient dietary intake, especially in exclusively breastfed infants. This review summarizes the biochemistry and pharmacokinetics of vitamin K, focusing on their role in the pathogenesis of late vitamin K deficiency bleeding (VKDB), including intracranial hemorrhage in early infancy. The limitations of conventional coagulation tests are discussed, highlighting the importance of functional biomarkers such as PIVKA-II (Proteins Induced by Vitamin K Absence or Antagonist-II) for the early detection of subclinical deficiency. Despite effective prophylaxis at birth, late VKDB cases still occur, likely due to declining vitamin K levels over time and nutritional factors. These findings underscore the need for prolonged vitamin K supplementation following adequate prophylaxis at birth, particularly to protect high-risk newborns from late VKDB. Strategies may include vitamin K-containing multivitamin supplementation in preterm infants, as well as daily oral vitamin K supplementation (150 µg/day) in exclusively breastfed infants, in order to ensure adequate vitamin K status during early infancy.

## 1. Introduction

Vitamin K (VK) is a fat-soluble vitamin that plays a crucial role as a cofactor for γ-glutamyl carboxylase (GGCX), an enzyme responsible for the post-translational modification of vitamin K-dependent proteins [1,2]. This modification enables the conversion of glutamate residues into γ-carboxyglutamate (Gla), a process essential for the biological activation of these proteins [2]. It represents an essential bioactive compound required for optimal body function.

**Figure 1:**
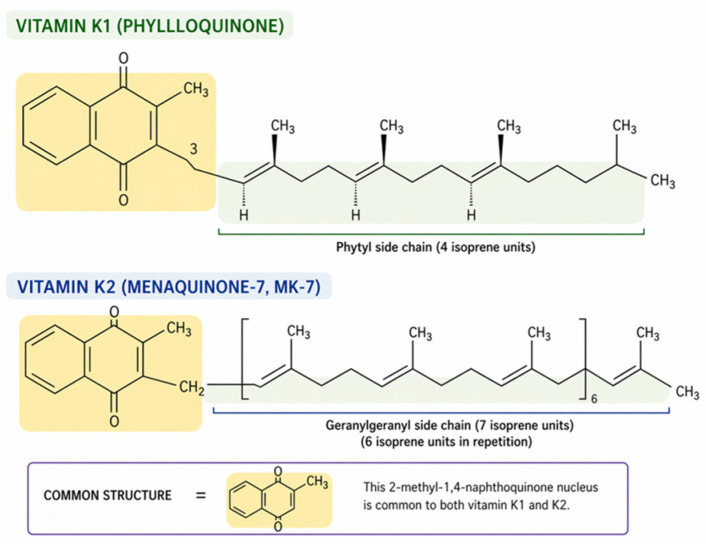
Chemical structures of vitamin K1 and vitamin K2. Both vitamin K1 (phylloquinone) and vitamin K2 (menaquinone-7) share a common 2-methyl-1,4-naphthoquinone nucleus (highlighted in yellow), which is essential for their biological role in -carboxylation reactions. The isoforms differ primarily in the length and saturation of their isoprenyl side chains: vitamin K1 contains a largely saturated phytyl chain (4 units), whereas vitamin K2 (MK-7) features a longer, fully unsaturated geranylgeranyl chain (7 units). These structural variations dictate the distinct pharmacokinetic profiles and tissue distributions of the two forms.

Vitamin K exists in two main forms: phylloquinone (vitamin K1), derived from plant sources, and menaquinones (vitamin K2), a group of compounds synthesized by bacteria [3]. All forms share a common structure, 2-methyl-1,4-napthoquinone, which is necessary for participation in carboxylation, but differ in the number and degree of saturation of isoprenyl side chains (Figure 1) [4].

According to the side-chain structure, menaquinones are currently divided into 10 isoforms (MK-4 to MK-13) [5], among which MK-4 and MK-7 are common and the most relevant in human physiology.

Vitamin K1 is preferentially retained in the liver to assist carboxylation of clotting factors [6]. Phylloquinone is the primary dietary form, found in green leafy vegetables and plant oils, whereas menaquinones are present in fermented foods such as cheese and natto. Although gut microbiota can synthesize menaquinones, their contribution to overall vitamin K status in humans appears limited [7].

## 2. Biological Functions of Vitamin K

Vitamin K is essential for the activation of a group of proteins collectively known as Gla proteins. These include key coagulation factors (II, VII, IX, X) and anticoagulant proteins (C, S, Z), as well as proteins involved in bone metabolism and vascular health, such as osteocalcin and matrix Gla protein [3,8].

Only the carboxylate forms of these proteins are biologically active and promote a health profile like hemostasis. Therefore, vitamin K deficiency leads to the production of inactive precursor proteins, compromising physiological processes such as hemostasis.

Beyond coagulation, vitamin K—particularly menaquinones such as MK-7—has been implicated in regulating osteoporosis, atherosclerosis, cancer and inflammatory diseases without risk of negative side effects or overdosing [5,9].

γ-Glutamyl carboxylase is the only identified enzyme that uses vitamin K as a cofactor in humans. This protein catalyzes the oxidation of VK hydroquinone to convert specific glutamate residues to γ-carboxyglutamate residues in VK-dependent proteins, which are involved in various essential biological processes and diseases [2].

All vitamin k dependent proteins are known as Gla proteins (from gamma-carboxyglutamic acid). They can be subclassified into a few categories according to their most well-known effects. Four proteins primarily have effects on connective tissue mineralization or have a closely related role (four are well-documented Gla proteins: matrix Gla protein, osteocalcin, Gla-rich protein, and nephrocalcin). If the periostin is a Gla protein, is disputable. Among them, four are transmembrane receptors (proline-rich Gla proteins), one is a growth-factor-like signaling molecule, one binds to hyaluronic acid in the extracellular matrix, and another is gamma-glutamyl carboxylase itself [10].

**Figure 2:**
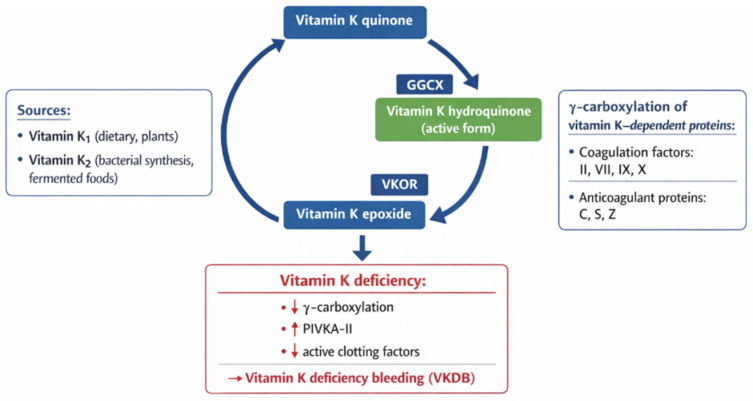
Vitamin K cycle and its role in γ-carboxylation and coagulation. Vitamin K acts as a cofactor for γ-glutamyl carboxylase (GGCX), enabling the activation of vitamin K-dependent proteins. The vitamin K cycle involves conversion between quinone, hydroquinone, and epoxide forms, mediated by GGCX and vitamin K epoxide reductase (VKOR). Vitamin K deficiency impairs γ-carboxylation, leading to increased levels of PIVKA-II and reduced activity of clotting factors, ultimately resulting in vitamin K deficiency bleeding (VKDB).

The vitamin K cycle and its role in γ-carboxylation and coagulation are summarized in Figure 2.

## 3. Vitamin K Deficiency in Neonates

Insufficient levels of vitamin K can lead to hematological complications due to impaired production of active coagulation molecules [11,12]. Newborns are particularly vulnerable to vitamin K deficiency, which may result in vitamin K deficiency bleeding (VKDB), a condition ranging from mild bleeding to life-threatening intracranial hemorrhage [13].

Several factors contribute to this vulnerability: limited trans-placental passage from mother to fetus with a gradient of 40:1 [14], presence in the umbilical cord with very small quantities (<0.02 ng/mL), lower concentrations in the maternal milk (2 µg/L) [4], immature liver function, short half-life a quick elimination of the deposits [15,16]. Therefore, neonatal synthesis of vitamin K-dependent clotting factors is significantly reduced [17].

## 4. Intracranial Bleeding in Early Infancy

Intracranial bleeding in early infancy is a clinical presentation rather than a single defined syndrome and may arise from a heterogeneous group of conditions, including prematurity-related germinal matrix/intraventricular hemorrhage, birth trauma, hypoxic–ischemic injury, vascular malformations, thrombocytopenia, congenital coagulation disorders, liver disease, and acquired coagulopathies such as vitamin K deficiency bleeding (VKDB) [18,19,20,21]. Genetic causes are also increasingly recognized, particularly in infants with structural vascular fragility or cerebrovascular malformations, such as COL4A1-related disease, whereas environmental and developmental contributors include prematurity, instrumental delivery, cholestasis, antibiotic exposure, and lack or failure of vitamin K prophylaxis [18,20].

In term infants, intracranial hemorrhage is less common than in very preterm neonates, but it remains a major cause of mortality and long-term neurodevelopmental impairment, including cerebral palsy, epilepsy, cognitive delay, and sensory deficits [19,20]. In late VKDB, intracranial hemorrhage is particularly relevant because it represents the most feared presentation and may account for approximately 30–60% of cases, with a substantial proportion of survivors showing neurological sequelae [12,22,23]. Clinical manifestations are often nonspecific at onset, including irritability, poor feeding, vomiting, pallor, lethargy, bulging fontanelle, seizures, or apnea, so diagnosis may be delayed until bleeding is already severe [22].

Among the many causes of intracranial bleeding in infancy, vitamin K deficiency is especially critical because it is both biologically plausible and potentially preventable. Vitamin K is required for the gamma-carboxylation and activation of coagulation factors II, VII, IX, and X, as well as proteins C and S; therefore, deficiency produces a systemic hemostatic defect that may remain clinically silent until a catastrophic bleeding event occurs [1,2,19]. In contrast with focal structural causes of hemorrhage, VKDB can involve otherwise healthy infants and may develop rapidly when low stores at birth, exclusive breastfeeding, impaired absorption, or hepatobiliary disease reduce effective vitamin K availability [13,19,24]. This makes vitamin K prophylaxis uniquely important among modifiable factors contributing to early infant intracranial bleeding.

Management of intracranial hemorrhage in infancy depends on the underlying etiology and severity. In suspected or confirmed VKDB, treatment includes prompt administration of vitamin K together with supportive replacement of coagulation factors, typically with fresh frozen plasma or, in selected life-threatening cases, prothrombin complex concentrate, in addition to neurocritical care and neurosurgical evaluation when indicated [19,23]. However, vitamin K should not be considered an adjuvant able to potentiate unrelated pharmacological therapies for intracranial hemorrhage; rather, its role is etiologic and corrective, because it directly reverses the acquired coagulopathy when deficiency is the driver of bleeding. Table 1 summarizes these concepts to improve clinical framing of the topic.

## 5. Biochemical Markers of Vitamin K Status

### 5.1. Challenges in Assessing Vitamin K Status

The assessment of vitamin K status in humans remains challenging, as measured levels can vary significantly depending on the analytical method used for detection [13].

Direct measurement of serum vitamin K can provide an indication of its circulating levels, but it does not necessarily reflect its utilization in target tissues. Accurate quantification is technically challenging due to the vitamin’s very low endogenous concentration, typically in the range of parts per billion. Analytical methods therefore rely on highly sensitive techniques such as high-performance liquid chromatography (HPLC) with fluorescence detection or liquid chromatography–mass spectrometry (LC–MS). These techniques are widely used for the analysis of vitamin K content in food and allow the quantification of individual vitamin K isoforms. For instance, a European standard method [30] has been established for the determination of vitamin K1 using HPLC; however, no officially recognized method is currently available for vitamin K2. Moreover, these techniques are resource-intensive and often not accessible in low-resource settings [31]. Thus, serum vitamin K is not routinely used in clinical practice due to analytical complexity and the fact that it reflects recent dietary intake rather than tissue utilization.

### 5.2. Limitations of Conventional Coagulation Tests

The diagnosis of VKDB is commonly indicated by a prolonged activated partial thromboplastin time (APTT) and prothrombin time (PT). VKDB is characterized by a PT international normalized ratio (INR) ≥ 4 or a value > 4 times the normal values in the presence of a normal platelet count and fibrinogen level. The diagnosis is confirmed based on the increased levels of PIVKAs and a rapid normalization of coagulation parameters after vitamin K administration [32].

Global coagulation assays, such as prothrombin time (PT) and activated partial thromboplastin time (aPTT), are widely used but lack sensitivity for detecting early vitamin K deficiency. PT typically becomes prolonged only when prothrombin levels fall below approximately 50% of normal [7].

During a bleeding event, global coagulation assays provide an indication of vitamin K involvement, which is because the PT reflects the combined activities of three vitamin K dependent proteins (factors II, VII and X) and two non-vitamin K dependent proteins (factor V and fibrinogen) [33]. However, reductions in fibrinogen activity need to be marked before they prolong a PT (<1.0 g/L), and factor V deficiency is extremely rare. The APTT is less sensitive to vitamin K status than the PT because a normal APTT requires the presence of the following coagulation factors: I, II, V, VIII, IX, X, XI and XII—a high proportion of which are not vitamin K dependent [34].

### 5.3. PIVKA-II as a Functional Biomarker

To detect vitamin K deficiency, one effective method is the measurement of Proteins Induced by vitamin K Absence or Antagonist-II (PIVKA-II), a functional biomarker that is typically undetectable in healthy adults and formula-fed infants [25].

PIVKA-II refers to an abnormal form of prothrombin produced in conditions of vitamin K deficiency or when vitamin K antagonists (such as warfarin) impair its function [35]. Specifically, PIVKA-II lacks γ-carboxylation, a crucial post-translational modification required for the biological activity of vitamin K-dependent clotting factors [25].

PIVKA-II is widely used as a sensitive marker for detecting subclinical vitamin K deficiency and has been shown to correlate well with the severity of the deficiency [28]. Elevated PIVKA-II levels are observed in liver dysfunction, including cirrhosis and hepatocellular carcinoma, as a consequence of impaired vitamin K utilization and reduced synthesis of clotting factors [9]. Elevated levels may also indicate vitamin K deficiency in at-risk populations, such as neonates or individuals undergoing prolonged antibiotic therapy that disrupts the gut microbiota [28].

While serum vitamin K concentrations reflect current tissue stores, PIVKA-II provides information on recent hepatic utilization of the vitamin. In clinical interpretation, low or undetectable serum vitamin K levels indicate inadequate tissue stores, whereas elevated PIVKA-II levels suggest that marginal stores have reached a threshold at which hepatic γ-carboxylation of prothrombin (factor II) is compromised [36].

Importantly, PIVKA-II is particularly useful for identifying subclinical vitamin K deficiency that may not be detected by standard coagulation tests. Indeed, elevated PIVKA-II levels precede changes in prothrombin time (PT) [25]. PIVKA-II concentrations above 0.05 AU/mL are considered indicative of early vitamin K insufficiency and have been associated with VKDB in infants [37,38].

For the diagnosis of VKDB, PIVKA-II has clinical utility as a retrospective biomarker, since laboratory samples are often collected after vitamin K administration [38]. Similarly, in cases of suspected non-accidental injury, PIVKA-II measurement can help determine whether bleeding events may be attributable to underlying vitamin K deficiency.

It is therefore important to distinguish that serum vitamin K reflects current stores, whereas PIVKA-II reflects hepatic functional utilization. Measurement of PIVKA-II is typically performed using immunoassays such as ELISA or chemiluminescent assays, with newer methods providing improved analytical sensitivity [39].

Serum PIVKA-II levels after birth show only a weak correlation with gestational age. Bovill et al. reported an increase in PIVKA-II positivity rates with advancing gestational age (5.3% at <34 weeks, 5.7% at 34–38 weeks, and 9.9% at ≥38 weeks), although these differences were not statistically significant [40]. In contrast, Santorino et al. identified preterm birth as an independent risk factor for elevated PIVKA-II levels [41].

PIVKA-II levels in neonates may also be influenced by additional physiological and environmental factors. Changes in the intrauterine environment around 37 weeks of gestation may affect PIVKA-II production. A review comparing term and prolonged pregnancies described placental microscopic changes, including syncytiotrophoblast nuclear aggregation and reduced villous vascularity, associated with impaired trophoblast transport and increased oxidative stress, potentially reflecting placental aging or functional decline [42].

Consistently, a study comparing oxidative stress markers in placentas from 37 to 39 weeks versus ≥41 weeks of gestation demonstrated significantly higher oxidative stress in the latter group, supporting the concept of placental aging or damage beyond 37 weeks [43]. Furthermore, immunohistochemical expression of 8-hydroxy-2′-deoxyguanosine (8-OHdG), a marker of oxidative DNA damage, has been shown to increase significantly with gestational age in normal placental tissue [44].

A decline in placental function after 37 weeks may reduce transplacental transfer of vitamin K to the fetus, contributing to the physiological vitamin K deficiency observed in term newborns. In a study evaluating placental transfer of vitamin K1, the maternal-fetal gradient of endogenous vitamin K1 concentrations was lower in mid-trimester (14-fold) compared with term (18-fold), suggesting reduced fetal availability at later gestational stages [45].

In adults, a PIVKA-II level ≥ 5000 mAU/mL is considered indicative of overt vitamin K deficiency, based on ranges observed in stable patients receiving warfarin therapy (targeting a prothrombin time international normalized ratio ≥ 1.5), which typically range from 6.9 to 99.5 AU/mL [26,27]. In experimental settings, PIVKA-II levels ≥1000 mAU/mL represent unequivocally abnormal undercarboxylated prothrombin concentrations, indicating vitamin K deficiency [26,46].

In neonates, however, lower thresholds are applied. A PIVKA-II level ≥ 200 mAU/mL has been proposed as a cutoff for latent vitamin K deficiency [47], largely due to the limited sensitivity (≥200 mAU/mL) of earlier ELISA-based assays [48,49]. More recently, chemiluminescent enzyme immunoassays (CLEIA), with a sensitivity of ≥1 mAU/mL, have become the preferred method for PIVKA-II measurement [50].

Using these more sensitive techniques, a cutoff value of ≥50 mAU/mL has been proposed for screening purposes in high-risk adult populations, such as those at risk for hepatocellular carcinoma [51]. In neonates, PIVKA-II levels above 50 mAU/mL—most commonly ranging between 200 and 1000 mAU/mL—have been associated with VKDB [37,52].

Notably, Perrone et al. demonstrated higher PIVKA-II levels in exclusively breastfed infants who did not receive prolonged vitamin K supplementation compared with those who received appropriate prophylaxis after birth, supporting the importance of continued supplementation in preventing early and classic forms of vitamin K deficiency bleeding [52].

## 6. Pharmacokinetics and Implications for Late VKDB

Vitamin K pharmacokinetics in infants show a decline in plasma levels of approximately 1000–2000 ng/mL immediately after parenteral administration at birth to approximately 0.16 ng/mL by the 6th week of life, particularly in exclusively breastfed infants. This decline coincides with low vitamin K content in breast milk (~1 ng/mL) and may lead to subclinical deficiency, as indicated by elevated PIVKA-II levels [47,53].

Although intramuscular prophylaxis at birth is highly effective to prevent early and classic form of VKDB, cases of late VKDB continue to be reported [54]. This suggests that current preventive strategies may not fully address all at-risk populations.

Given the rarity of VKDB, randomized clinical trials are not feasible, and surrogate markers such as PIVKA-II remain essential for evaluating preventive strategies.

In addition to the decline in circulating vitamin K levels over time, several physiological factors influence pharmacokinetics in early infancy. Vitamin K absorption is a bile-dependent process, and in neonates, bile acid production and secretion are still immature compared with older children and adults [55]. In addition, pancreatic enzyme activity and intestinal fat absorption are reduced in the neonatal period [56]. These maturational differences may limit the intestinal absorption of orally administered vitamin K, particularly in the first weeks of life, and contribute to the higher interindividual variability in systemic exposure observed after oral supplementation.

In contrast, older children and adults have fully developed bile acid pools, efficient micellar solubilization of fat-soluble vitamins, and a mature intestinal mucosa, resulting in more predictable and generally higher bioavailability of vitamin K after oral administration. Furthermore, hepatic uptake, recycling via the enterohepatic circulation, and vitamin K-dependent carboxylation pathways are more stable and functionally efficient beyond the neonatal period [4], contributing to more consistent pharmacokinetic profiles.

Furthermore, vitamin K is transported in plasma primarily via lipoproteins, including chylomicrons and low-density lipoproteins. Neonates exhibit differences in lipoprotein metabolism compared to older children and adults [57,58], which may affect the distribution and tissue availability of vitamin K. This aspect may be particularly relevant in preterm infants, who often display altered lipid metabolism.

Another important consideration is the relatively short half-life of vitamin K-dependent clotting factors, especially factor VII, which has a half-life of approximately 4–6 h [59]. This rapid turnover means that even transient reductions in vitamin K availability can quickly translate into impaired coagulation capacity. Consequently, maintaining stable vitamin K levels over time is essential to ensure continuous γ-carboxylation of these proteins.

In addition, the hepatic storage capacity for vitamin K in neonates is limited [60]. Unlike adults, who can rely on hepatic reserves during periods of low intake, newborns depend more heavily on continuous external supply. This further supports the concept that a single prophylactic dose at birth may not be sufficient to maintain adequate vitamin K status throughout early infancy.

Overall, these pharmacokinetic considerations highlight the dynamic nature of vitamin K metabolism in neonates and reinforce the rationale for prolonged supplementation strategies aimed at preventing late VKDB.

## 7. Role of Gut Microbiota and Antibiotic Exposure in Vitamin K Status

Vitamin K availability in early infancy is also influenced by the development of gut microbiota and exposure to antibiotics.

The role of gut microbiota on vitamin K status in early life remains a topic of ongoing investigation. Menaquinones (vitamin K2), synthesized by intestinal bacteria, have been traditionally considered a potential endogenous source of vitamin K, particularly in adults, in whom a mature and stable gut microbiota—rich in anaerobic species such as *Bacteroides* and members of the *Firmicutes phylum*—can contribute substantially to overall vitamin K2 production [29]. However, in neonates, this contribution appears to be minimal due to the immaturity of the gut microbiome [22].

At birth, the neonatal intestine is largely sterile, and microbial colonization occurs progressively over the first weeks and months of life [61]. This represents a fundamental difference compared with adults, in whom the gut microbiota is already fully established and metabolically active in vitamin K cycling. This delayed establishment of a stable microbiota limits the endogenous production of menaquinones during a critical window in which infants are already vulnerable to vitamin K deficiency. Furthermore, the bacterial species responsible for menaquinone synthesis are not predominant in the early neonatal microbiome, further reducing this potential source.

Notably, there is no evidence that the maternal gut microbiota directly colonizes the fetus in utero [62]; therefore, fetal exposure to microbiota-derived vitamin K is negligible. Maternal influence on neonatal vitamin K status is instead primarily indirect, occurring through placental transfer of phylloquinone, which is itself limited [14], and through breast milk, which contains low concentrations of vitamin K [63]. Thus, while maternal microbiota may indirectly influence maternal vitamin K metabolism, it does not represent a direct source of vitamin K for the fetus or neonate.

Antibiotics therapy, which is relatively common in neonatal care, particularly in preterm infants or in cases of suspected infection, can also affect vitamin k status. Broad-spectrum antibiotics can significantly disrupt gut microbial composition [64], reducing populations of vitamin K-producing bacteria and potentially contributing to deficiency. Clinical observations have shown that prolonged antibiotic therapy is associated with increased levels of PIVKA-II [65], suggesting impaired vitamin K availability and utilization.

Taken together, these findings suggest that the contribution of gut microbiota to vitamin K status in early infancy is limited and highly variable. Moreover, antibiotic exposure represents an additional risk factor that may exacerbate subclinical deficiency. These considerations support the need for continued vitamin K supplementation strategies that do not rely on endogenous production, particularly in high-risk neonatal populations.

## 8. Special Considerations: Gestational Age and Placental Transfer

Emerging evidence suggests that vitamin K status may vary according to gestational age. Some studies indicate that preterm infants may not necessarily be at higher risk than term infants, possibly due to differences in placental transfer dynamics. Placental aging with advancing gestational age is associated with morphological changes that could impact the transfer of nutrients. This reduction in placental function is also associated with increased evidence of oxidative stress, which may reflect progressive placental dysfunction [43]. A study comparing oxidative stress biomarkers between placentas at 37–39 weeks and those at 41–43 weeks reported a significant increase in oxidative stress in the latter group, suggesting placental aging or damage after 37 weeks of gestation [44]. Moreover, a study examining the expression of DNA damage markers according to gestational age in normal placentas found that the immunohistochemical expression of 8-hydroxy-2′-deoxyguanosine, a recognized reliable marker of lipoperoxidation, significantly increased with advancing gestational age [45].

Thus, it has been hypothesized that the aforementioned decline in placental function around term of gestation may lead to a reduced placental transfer of vitamin K to the fetus, thereby contributing to the physiological vitamin K deficiency observed in term newborns [66]. Supporting this hypothesis, a study evaluating the placental transfer of vitamin K through maternal, fetal, and neonatal serum samples showed that the mean maternal–fetal gradient of endogenous vitamin K concentrations at mid-trimester was lower than that observed at term, which is consistent with a reduced transfer efficiency later in pregnancy [27].

Overall, placental aging and the associated increase in oxidative stress after 37 weeks of gestation may impair the transfer of essential nutrients, including vitamin K, thereby contributing to a physiological deficiency in term newborns.

## 9. Why Does Late VKDB Still Occur Despite Prophylaxis?

Despite the widespread implementation of vitamin K prophylaxis at birth, cases of late VKDB continue to be reported, raising important questions about the adequacy of current preventive strategies. Several factors may contribute to this phenomenon, involving pharmacokinetics, nutritional factors, and individual variability.

First, the pharmacokinetic profile of vitamin K suggests that a single intramuscular dose at birth may sustained adequate levels throughout early infancy. Studies have shown that plasma vitamin K concentrations decline progressively within the first weeks of life, particularly in exclusively breastfed infants, reaching low levels by 6–8 weeks [47]. This decline coincides with the period of highest risk for late VKDB.

Second, breast milk contains relatively low concentrations of vitamin K compared to formula milk, which is routinely supplemented [53]. As a result, exclusively breastfed infants remain at higher risk of subclinical deficiency, even after appropriate prophylaxis at birth. This is supported by higher PIVKA-II levels observed in breastfed infants who did not receive additional supplementation [37,52,54].

Third, interindividual variability in vitamin K metabolism and absorption may play a role. Factors such as immature hepatic function, differences in bile acid secretion, and variability in intestinal absorption can influence vitamin K bioavailability in early life. In addition, conditions such as cholestasis or unrecognized liver dysfunction may impair vitamin K utilization, increasing the risk of bleeding [66,67].

Another potential contributing factor is the limited placental transfer of vitamin K. The marked maternal–fetal gradient, combined with possible reductions in placental efficiency at term, may result in suboptimal neonatal stores at birth. Emerging evidence suggests that placental aging and oxidative stress beyond 37 weeks of gestation could further impair nutrient transfer, including vitamin K, thereby contributing to early postnatal vulnerability [68].

Furthermore, current monitoring strategies are inadequate for detecting early or subclinical deficiency. Conventional coagulation tests lack sensitivity, and vitamin K levels are difficult to measure. Although PIVKA-II is a promising biomarker, it is not routinely used in clinical practice, limiting the ability to identify infants at risk before clinical manifestations occur.

Finally, the rarity of VKDB makes it difficult to conduct randomized controlled trials to optimize prophylaxis regimens. As a result, current recommendations are largely based on observational data and surrogate endpoints.

**Figure 3:**
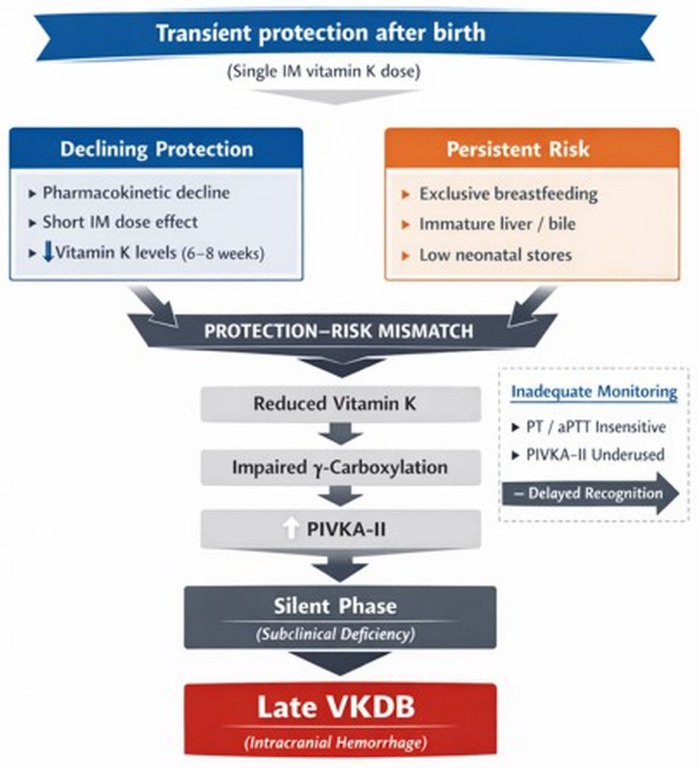
Protection-risk mismatch underlying late vitamin K deficiency bleeding (VKDB). A single intramuscular dose of vitamin K at birth provides transient protection, but its effect progressively declines during early infancy. In parallel, persistent risk factors—including exclusive breastfeeding, developmental immaturity affecting vitamin K metabolism, and low neonatal stores due to limited placental transfer-maintain a sustained vulnerability to deficiency. This temporal mismatch between declining protection and ongoing risk results in reduced effective vitamin K availability. A clinically silent phase of subclinical deficiency, characterized by increased PIVKA-II levels, precedes impaired γ-carboxylation of vitamin K-dependent proteins and culminates in late VKDB, often presenting as severe bleeding such as intracranial hemorrhage. Inadequate monitoring strategies further limit early detection.

Taken together, these factors, as illustrated in Figure 3, indicate that late VKDB is not merely a failure of prophylaxis, but rather reflects a complex interplay between pharmacokinetics, nutritional factors, developmental physiology, and limitations in current monitoring strategies. A better understanding of these mechanisms may help refine prevention strategies, potentially including tailored supplementation approaches and improved biomarker-guided monitoring.

## 10. How Much Vitamin K Is Necessary to Prevent Late Bleeding?

A key issue in the prevention of late VKDB in neonates is the definition of adequate vitamin K intake. This remains challenging, as current requirements are largely based on estimations and on the assumption that vitamin K administration at birth provides sufficient stores to sustain physiological needs over time [53].

Typical recommended vitamin K intake in North America ranges widely, from 50 up to 600 μg/day for vitamin K1 and from 5 to 600 μg/day for vitamin K2 [39]. According to the National Academy of Medicine (NAM), previously known as the Institute of Medicine, the recommended daily intake is 120 µg/day for adult men and 90 µg/day for adult women. The World Health Organization (WHO) and the Food and Agriculture Organization (FAO) suggest lower values—65 µg/day for men and 55 µg/day for women—based on an estimated requirement of 1 µg/kg/day. The European Commission has established a reference intake of 75 µg/day for adults [39,68,69,70].

In neonates, however, vitamin K requirements have not been fully standardized, and recommendations vary considerably across countries and scientific societies [71,72]. This lack of consensus reflects both the limited evidence available and the physiological peculiarities of the neonatal period.

Exclusively breastfed newborns are particularly vulnerable, as breast milk contains only minimal amounts of vitamin K and is insufficient to meet neonatal requirements [73]. Consequently, ongoing supplementation may be necessary to ensure adequate intake and to reduce the risk of deficiency.

Prophylaxis with 1 mg of vitamin K at birth, followed by oral administration of 150 µg daily from 2 to 14 weeks of life in exclusively breastfed infants, has been associated with the lowest levels of PIVKA-II at 3 months of age, indicating effective protection against late VKDB.

## 11. Conclusions

While the role of Vitamin K as a cofactor in γ-carboxylation is well-established, its clinical management in neonates remains a moving target. The clinical utility of PIVKA-II as a sentinel biomarker offers a window of opportunity for early intervention that traditional tests lack. However, diagnosis alone is insufficient. The continued occurrence of late VKDB underscores an urgent need to rethink neonatal care: we must move beyond immediate postnatal doses toward prolonged, standardized prophylaxis. Strategies may include vitamin K-containing multivitamin supplementation in preterm infants [52], as well as daily oral vitamin K supplementation (150 µg/day) in exclusively breastfed infants [54], in order to ensure adequate vitamin K status during early infancy.

## Figures and Tables

**Table 1:** Clinical settings of intracranial bleeding in early infancy, current therapies, and role of vitamin K.

Reference	Clinical Setting	Main Causes	Standard Management	Role of Vitamin K
[1,2,19]	VKDB	Inadequate vitamin K stores, exclusive breastfeeding, lack of prophylaxis, malabsorption	Vitamin K (IV/IM), fresh frozen plasma (FFP), or prothrombin complex concentrates (PCC) in severe cases	Causal therapy: restores γ-carboxylation of clotting factors and corrects coagulopathy
[18,20]	Intracranial hemorrhage unrelated to VK deficiency	Birth trauma, vascular malformations, genetic coagulation disorders	Supportive care, neurosurgical intervention, blood products as needed	No direct therapeutic role, unless concomitant subclinical vitamin K deficiency is present
[23,25,26]	Subclinical vitamin K deficiency (elevated PIVKA-II, no bleeding)	Exclusive breastfeeding, declining vitamin K levels after birth	Oral vitamin K supplementation	Preventive role: avoid progression to overt VKDB
[13,24,27]	Cholestasis or malabsorption-associated bleeding	Impaired bile flow, liver disease, biliary atresia	Parenteral vitamin K, management of underlying disease, possible surgical intervention	Essential therapy: oral vitamin K ineffective; parenteral administration required
[28,29]	Antibiotic-associated coagulopathy	Disruption of gut microbiota reducing menaquinone production	Vitamin K supplementation, review of antibiotic therapy	Adjunctive role: restores vitamin K availability and coagulation function

## Data Availability

No new data were created or analyzed in this study. Data sharing is not applicable to this article.

## Abbreviations

The following abbreviations are used in this manuscript:

VK	Vitamin K
VK1	Phylloquinone (Vitamin K1)
VK2	Menaquinones (Vitamin K2)
MK	Menaquinone
GGCX	γ-Glutamyl carboxylase
Gla	γ-carboxyglutamate
VKDB	Vitamin K deficiency bleeding
PIVKA-II	Proteins Induced by Vitamin K Absence or Antagonist-II
PT	Prothrombin time
aPTT	Activated partial thromboplastin time
